# Self-management interventions for Type 2 Diabetes: systematic review protocol focusing on patient workload and capacity support

**DOI:** 10.12688/wellcomeopenres.17238.1

**Published:** 2021-10-11

**Authors:** Peter Hanlon, Iona Bryson, Holly Morrison, Qasim Rafiq, Kasey Boehmer, Michael R Gionfriddo, Katie Gallacher, Carl May, Victor Montori, Jim Lewsey, David A McAllister, Frances S Mair

**Affiliations:** 1 Institute for Health and Wellbeing, University of Glasgow, Glasgow, UK; 2 Knowledge and Evaluation Research Unit, Mayo Clinic, Rochester, USA; 3 School of Pharmacy, Duquesne University, Pittsburgh, USA; 4 London School of Hygiene and Tropical Medicine, London, UK

**Keywords:** Diabetes, type 2 diabetes mellitus, self-management, treatment burden, patient capacity

## Abstract

Introduction:

People living with type 2 diabetes undertake a range of tasks to manage their condition, collectively referred to as self-management. Interventions designed to support self-management vary in their content, and efficacy. This systematic review will analyse self-management interventions for type 2 diabetes drawing on theoretical models of patient workload and capacity.

Methods and analysis:

Five electronic databases (Medline, Embase, CENTRAL, CINAHL and PsycINFO) will be searched from inception to 27th April 2021, supplemented by citation searching and hand-searching of reference lists. Two reviewers will independently review titles, abstracts and full texts. Inclusion criteria include Population: Adults with type 2 diabetes mellitus; Intervention: Randomised controlled trials of self-management support interventions; Comparison: Usual care; Outcomes: HbA1c (primary outcome) health-related quality of life (QOL), medication adherence, self-efficacy, treatment burden, healthcare utilization (e.g. number of appointment, hospital admissions), complications of type 2 diabetes (e.g. nephropathy, retinopathy, neuropathy, macrovascular disease) and mortality; Setting: Community. Study quality will be assessed using the Effective Practice and Organisation of Care (EPOC) risk of bias tool. Interventions will be classified according to the EPOC taxonomy and the PRISMS self-management taxonomy and grouped into similar interventions for analysis. Clinical and methodological heterogeneity will be assessed within subgroups, and random effects meta-analyses performed if appropriate. Otherwise, a narrative synthesis will be performed. Interventions will be graded on their likely impact on patient workload and support for patient capacity. The impact of these theoretical constructs on study outcomes will be explored using meta-regression.

Conclusion

This review will provide a broad overview of self-management interventions, analysed within the cumulative complexity model theoretical framework. Analyses will explore how the workload associated with self-management, and support for patient capacity, impact on outcomes of self-management interventions.

Registration number:

PROSPERO CRD42021236980.

## Background

Type 2 diabetes is a long-term condition characterised by elevated blood glucose levels resulting from insulin resistance and relative insufficiency of insulin ^1^. Type 2 diabetes is common and rising in prevalence throughout the world, resulting in a considerable burden on individuals and healthcare systems ^2^. Complications such as macrovascular disease (e.g. myocardial infarction, peripheral vascular disease) and microvascular disease (e.g. nephropathy, retinopathy) can develop in people with type 2 diabetes and are responsible for considerable morbidity and mortality. However, the development and impact of complications can be limited by managing type 2 diabetes well. This includes controlling blood sugar levels (e.g. through diet and/or medication), managing risk factors (such as blood pressure and lipid levels), promoting behaviours such as exercise, and monitoring for development of complications ^3^. Controlling type 2 diabetes therefore carries a range of tasks and activities that people living with type 2 diabetes should undertake. These tasks are collectively described as ‘self-management’.

Supporting people with type 2 diabetes to self-manage their condition is a cornerstone of diabetes care. Interventions to support self-management include structured education, support for self-monitoring, lifestyle advice and support for behaviour change, and access to information and advice. Interventions aiming to support self-management in type 2 diabetes are highly variable in their aims, content, and level of support ^4^. Previous systematic reviews have synthesised evidence for self-management, either in general or focussing on specific modes of delivery (e.g. group-based education, telemedicine, or internet-based interventions), and have shown that self-management support can be effective in reducing glycated haemoglobin (HbA1c) in the short- to medium-term ^4, 5^. There is, however, considerable heterogeneity in the effectiveness of self-management support interventions ^4^. Moreover, the impact on long-term glycaemic control and on other outcomes (such as complications or quality of life) is inconsistent ^4, 5^. It is not clear what components or approaches to supporting self-management are optimal, although multi-component interventions with greater contact time appear to be most effective ^4^.

Self-management, by its nature, involves work on the part of the person living with type 2 diabetes. The impact of this work on wellbeing has been conceptualised as the *burden of treatment* ^6, 7^. Burden of treatment is a slightly broader concept than workload, and encompasses the ‘work’ involved, on the part of a person living with a long-term condition, to manage their condition and the resultant impact on that person’s life. The work of self-management will vary from person to person depending on disease-related factors as well as the way healthcare services are delivered and configured and the wider context. Closely related to this, is the ability of a person to take on the work involved in managing their condition; referred to as patient capacity. Capacity is influenced by physical or mental morbidity, functional disability, personal attributes (e.g. literacy), social support, wider life workload (e.g. dependents), financial circumstances, and environment ^8^. Capacity varies from person to person, and a given workload of self-management will present different challenges to different individuals, therefore both workload and patient capacity need to be considered when designing interventions.

Capacity is centrally important in managing type 2 diabetes, as type 2 diabetes itself is associated with a range of factors which directly impact patient capacity. Type 2 diabetes is strongly associated with lower socioeconomic status, which is in turn associated with complications of diabetes ^9^. Multimorbidity, the presence of multiple long-term conditions, is common in type 2 diabetes ^10, 11^. This includes concordant (sharing similar risk factors, causes and management strategies) and discordant (distinct risk factors and management) long-term conditions ^12^. Frailty, a state of reduced physiological reserve, is also highly prevalent in people with type 2 diabetes, affecting between 10 and 25% of people over the age of 60, as well as some in middle-age ^13– 15^. People with severe mental illness are also more than twice as likely to develop type 2 diabetes as people without ^16^. Therefore, to be effective for the majority of people with type 2 diabetes, strategies to support self-management must accommodate factors which may reduce a person’s capacity to cope with the workload of self-management.

This review will set out to test two hypotheses based on an understanding of workload and capacity:

1. We hypothesise that interventions that increase or enhance capacity are more likely to improve outcomes
2. We hypothesise that increases in workload are more likely to improve outcomes where they occur alongside a corresponding increase in capacity support

These hypotheses will be tested by addressing the following aims:

1. To assess the ‘workload’ for patients, and support for patient capacity, of self-management support interventions for people with type 2 diabetes.
2. Quantify the impact of interventions on the management of type 2 diabetes (HbA1c, quality of life, burden of treatment, healthcare utilisation and mortality).
3. Explore which features of interventions, including the effect on workload and the patient’s capacity to self-manage, are associated with greater improvements in outcomes.

## Methods

### Theoretical constructs

**Figure 1.**
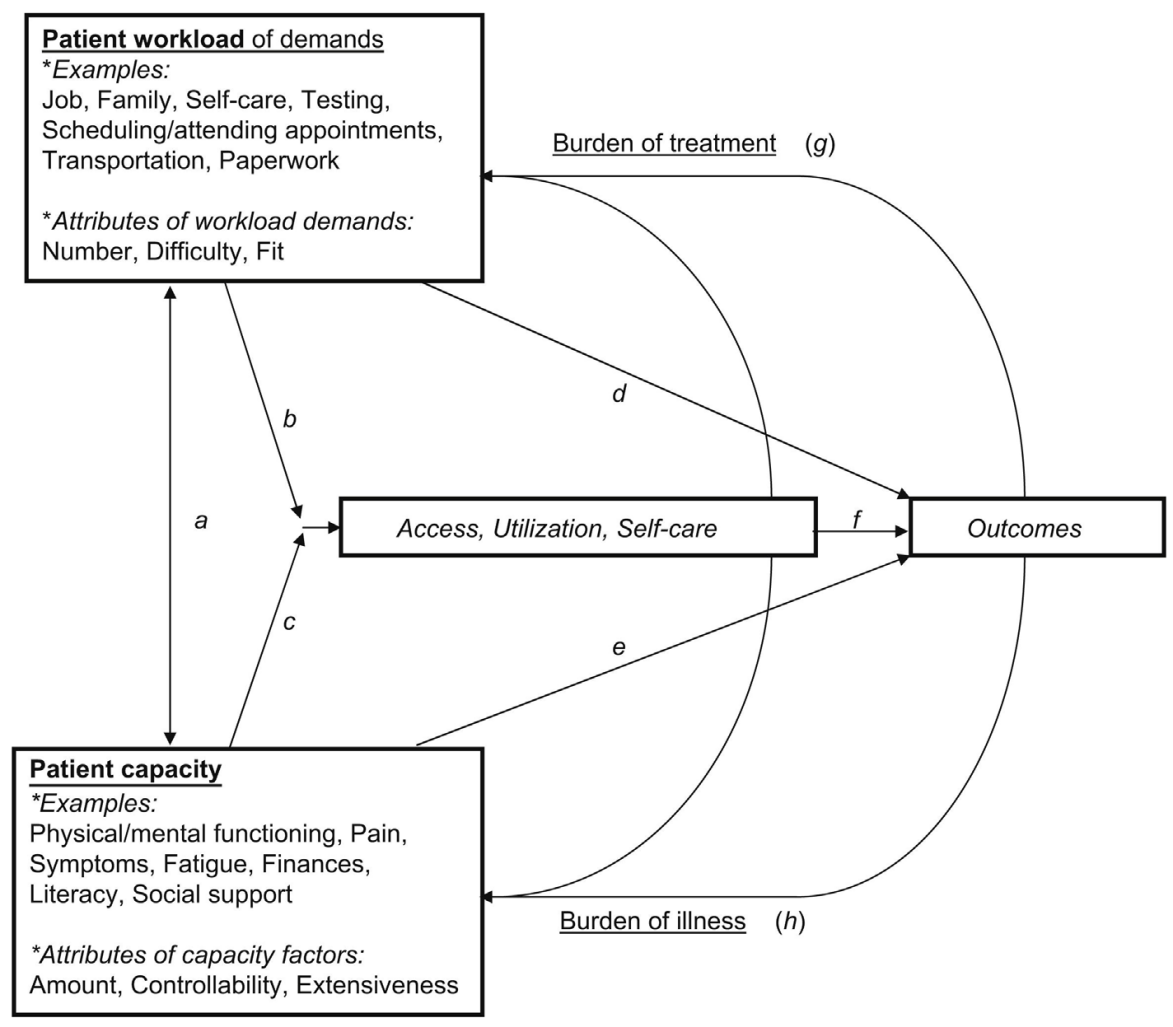
Cumulative complexity model. Reproduced with permission: Shippee ND, Shah ND, May CR, Mair FS, Montori VM. Cumulative complexity: a functional, patient-centred model of patient complexity can improve research and practice. Journal of clinical epidemiology. 2012 Oct 1;65(10):10415–1.

This review will aim to synthesise evidence from a broad range of self-management interventions, with a specific focus on workload and capacity. We will draw upon a range of interrelated theoretical models which conceptualise workload and burden of treatment and patient capacity, summarised in Box 2– Box 4 and Figure 1. These theoretical models include Normalisation Process Theory (describing the type of work involved in interventions) ^17, 18^, the Theory of Patient Capacity (commonly referred to by its acronym BREWS, which considers a range of aspects of capacity including Biography, Resources, Environment, realisation of Work, and Social networks) ^19^, Burden of Treatment Theory (which conceptualises how workload interacts with a person’s wider circumstances resulting in treatment burden) ^6^, and the Cumulative Complexity Model (which considers the interactions between the two constructs of workload and capacity) ^20^.

The Cumulative Complexity Model comprises two main constructs: workload and capacity. Workload describes the demands placed on a patient which includes the work of self-management (burden of treatment) as well as other wider life world demands (e.g. employment, caring for relatives). Capacity, on the other hand, describes a persons’ ability and available resources to handle a given workload. Cumulative Complexity Model implies that the balance between workload and capacity directly impacts a persons’ experience of health, their behaviours, and their interactions with healthcare. An imbalance of workload and capacity may lead to poor health-related outcomes, which may further reduce capacity and increase workload.

### Search strategy

Electronic databases will be searched using a combination of keywords and Medical Subject Headings (MeSH). We will search Medline, Embase, Cochrane Central Register for Controlled Trials (CENTRAL), and Cumulative Index to Nursing and Allied Health (CINAHL) and PsycINFO. Search terms for Medline are shown in Box 1 and will be adapted for the other databases based on variation in MeSH terms. The trials filter is taken from the Cochrane Handbook for Systematic Reviews and is designed to balance sensitivity and specificity.

**Box 1: Search terms**

        
**1. Exp Diabetes Mellitus/**

        
2. Diabet*.tw

        
**3. (NIDDM or T2DM or T2D).tw**

        
4. (non insulin* depend* or non insulin depend* or non insulin?depend* or non insulin?depend).tw.

        
**5. exp Diabetes Insipidus/**

        
6. diabet* insipidus.tw

        
**7. 1 or 2 or 3 or 4**

        
**8. 5 or 6**

        
**9. 7 not 8**

        
**10. exp Self Care/**

        
**11. exp Professional-Family Relations/**

        
**12. exp Professional-Patient Relations/**

        
**13. exp Health Education/**

        
**14. exp "Attitude of Health Personnel"/**

        
**15. exp Patient Education as Topic/**

        
**16. exp Self Efficacy/**

        
**17. exp "Activities of Daily Living"/**

        
**18. exp Self-Help Devices/**

        
19. ((Self adj2 (car* or manag* or help or administ* or monitor* or medicat*)) or self-car* or self-manag* or selfhelp or self-administ* or self-monitor* or self-medicat* or selfcar* or selfmanagement or selfhelp or selfadminist* or selfmonitor* or selfmedicat*).mp.

        
**20. expert patient.tw.**

        
**21. ((professional or clinician) adj2 development).tw.**

        
**22. (Confidence or self-efficacy).mp.**

        
23. ((patient or individual* or person* or client*) adj3 (remind* or feedback)).mp

        
24. ((home or environment* or living or assistive) adj2 (adaptation or modif* or equipment or technolog*)).mp.

        
**25. "action plan".tw.**

        
**26. "Care plan".tw**

        
**27. ((Peer or patient or emotional or social or psychosocial) adj1 (support or group)).mp.**

        
**28. 10 or 12 or 13 or 14 or 15 or 16 or 17 or 18 or 19 or 20 or 21 or 22 or 23 or 24 or 25 or 26 or 27**

        
**29. randomized controlled trial.pt.**

        
**30. controlled clinical trial.pt.**

        
**31. randomized.ab.**

        
**32. placebo.ab.**

        
**33. clinical trials as topic.sh**

        
**34. randomly.ab**

        
**35. trial.ti.**

        
**36. 29 or 30 or 31 or 32 or 33 or 34 or 35**

        
**37. exp animals/ not humans.sh.**

        
**38. 36 not 37**

        
**39. 9 and 28 and 38**

**Box 2: Theoretical Frameworks: Normalisation Process Theory**

**Table:** 

Theoretical framework for defining the work involved in the interventions. The components of the theory are outlined below with an explanation of how each applies to type 2 diabetes self-management.
**Normalisation Process Theory components:** **Coherence (Sense-making work):** *Understanding the different aspects of type 2 diabetes, gathering information with the support of others, forming an understanding of the implications of living with type 2 diabetes and applying these to individual circumstances.* **Cognitive participation (Relationship work):** *The work involved in identifying and engaging with others to support management of the condition (including professions and family or friend support). This includes arranging this support and negotiating the patient’s own contribution to this interaction.* **Collective action (Enacting work):** *This describes the effort and resources that are put into managing the condition. Includes carrying out tasks and living with the effects of treatments.* **Reflexive monitoring (Appraisal work):** *The work of reflecting on the tasks involved in self-management and evaluating whether to modify these.*

**Box 3: Theoretical frameworks: Theory of Patient Capacity (BREWS)**

**Table:** 

Theoretical framework for defining the capacity support of interventions. The components of the theory are outlined below with an explanation of how each applies to type 2 diabetes self-management.
**Theory of Patient Capacity components:** **Biography:** *Supporting the process of patients reframing their life to include the reality of living with type 2 diabetes.* **Resources:** *Providing or facilitating access to resources (e.g. knowledge, transportation, time, financial support) that allow patients to better utilise their own capacity for self-management.* **Environment:** *Improving the environment in which a patient is able to manage their condition.* **(realisation of) Work:** *Promotes success in the experience of managing the work of self-management.* **Social networks:** *Supports patients’ interaction with wider social networks and sources of support.*

**Box 4: Theoretical Frameworks: Burden of Treatment Theory**

**Table:** 

1: Mobilising capacity – how patients understand the work that needs to be done	
1.1: Agency	Things patients do to engage with health problems and with others. It encompasses the acceptance of the diagnosis and understanding and accepting their role.
1.2: Relational Network	The voluntary and obliged network through which patients express and distribute the tasks of care. This network may include family or health and other professionals.
1.3: Opportunity	How availability of services (for example based on geographical location) affects capacity.
1.4: Control over service	How organisations determine the content, structure and resources of services and how this affects capacity.
**2: Expressing capacity – how patients perform the work**	
2.1: Social Skill	Skills necessary to engage and mobilise the cooperation of others, and to negotiate controls placed on resources to help with the self-management workload.
2.2: Functional Performance	Degree to which the patient possesses the cognitive and material capacity to meet demands.
2.3: Structural resilience	How the patient’s network of support can be used to absorb, compensate and even thrive when things change.
2.4: Social Capital	How the patient’s social network can be used for gaining information and resources that help with care.
**3: Mobilising for delegated tasks – how patients sustain and modify their role**	
3.1: Sense Making (coherence)	The patient’s understanding of the tasks that make up their work, how they make sense of it, and how they plan based on this information.
3.2: Building and maintaining relational networks (cognitive participation)	How patients enrol, engage and maintain contacts in their support network.
3.3: Enacting delegated work (collective action)	The process of operationalising self-management including allocating and undertaking self-management activities and negotiating accountability for self-management tasks.
3.31: Material and cognitive practices to be done (interactional workability)	Whether self-management tasks are perceived to be workable, and the practical things patients do to operationalise self-management responsibilities.
3.33: Practical Help (skill set workability)	Having or learning the practical skills to carry out self-management work.
3.34: Exploitable resources (contextual integration)	Having or obtaining the resources to carry out self-management activities.
3.35: Confidence in outcomes (relational integration)	Factors that influence whether patients believe the self-management tasks are the right thing to do and have confidence in the outcomes of delegated tasks.
3.4: Reflexive monitoring	Appraisal of their role, and whether any reconfiguration would help.

### Inclusion criteria

***Population.*** Included studies should focus on people with type 2 diabetes. Participants should be adults ≥18 years. Studies with more specific age-ranges will also be eligible for inclusion.

***Intervention.*** To be eligible for inclusion, studies should assess interventions to support self-management of type 2 diabetes.

Self-management refers to all behaviours undertaken by an individual to live with and manage an established health condition ^21^. Definitions vary, however we will use the definition adopted by the United States National Academy of Medicine ^22^:

“ *Self-management is defined as the tasks that individuals must undertake to live with one or more chronic conditions. These tasks include having the confidence to deal with medical management, role management and emotional management of their conditions*.”

We draw a distinction between this definition and the more generic concept of ‘self-care’, which describes all behaviours an individual must undertake to maintain health (i.e. not specifically linked to a specific medical condition). Eligible studies must explicitly aim to support self-management of type 2 diabetes.

***Comparison.*** We will include studies comparing the intervention of interest to usual care/standard practice.

***Outcomes.*** The primary outcome of the review will be change in glycated haemoglobin (HbA1c) as a measure of glycaemic control.

Secondary outcomes will include health-related quality of life (QOL), medication adherence, self-efficacy, treatment burden, healthcare utilization (e.g. number of appointment, hospital admissions), complications of type 2 diabetes (e.g. nephropathy, retinopathy, neuropathy, macrovascular disease) and mortality.

***Study design.*** Randomised controlled trials (including cluster-randomised controlled trials). Pilot and feasibility studies will be excluded.

***Setting.*** Community or outpatient setting. Studies based in an inpatient setting will be excluded.

### Exclusion criteria

We will exclude articles not published in English. This is due to a lack of resources to allow translation of articles.

We will also exclude studies that are not peer-reviewed studies (e.g. conference abstracts or grey literature).

### Screening and data extraction

Records identified from each database will be exported to Endnote and duplicates removed. All titles and abstracts will then be collated using Distiller Systematic Review software (DistillerSR. Version 2.35. Evidence Partners; 2021. https://www.evidencepartners.com), which will be used for screening.

Two reviewers, working independently, will screen all titles and abstracts using a piloted form based on the inclusion and exclusion criteria detailed above. Disagreement over eligibility will be resolved by discussion involving a third reviewer. Where there is still disagreement over eligibility at the abstract stage, studies will be retained for full-text assessment.

Full texts of all potentially eligible articles will be assessed independently by two reviewers.

The following data will then be extracted from the included studies:

• Study identifiers (Author, year, journal, country)
• Study population (Mean age (sd), sex, ethnicity, socioeconomic status, inclusion and exclusion criteria)
• Setting (e.g. community, primary care, outpatient clinic)
• Intervention characteristics (description of the intervention as per the study authors, mode of delivery classified according the EPOC taxonomy ^23^, components of self-management support classified using the PRISMS taxonomy ^24^, duration of intervention (i.e. total time from onset to completion), frequency of self-management support activities.
• Comparison group (description of ‘usual care’)
• Outcomes (Data for all outcomes assessed, along with length of follow-up and mode of assessment)

### Risk of bias assessment

The risk of bias of included studies will be assessed using the Cochrane Effective Practice and Organisation of Care (EPOC) risk of bias tool. Risk of bias will be assessed at the outcome level.

## Analysis and synthesis

### Aim 1: Workload and capacity support of included interventions

Our assessment of the workload of interventions, and the degree of support for patient capacity, will be carried out in two phases: descriptive coding of the interventions, and quantification of workload and capacity.

***Descriptive coding of self-management components, workload and capacity.*** The first, descriptive, stage will draw upon existing theoretical frameworks for self-management, workload and patient capacity. Reviewers extracting data will summarise the description of the intervention. They will then classify the self-management components of each intervention described in the included trials using the PRISMS self-management taxonomy ( Table 1) ^24^. The PRISMS self-management taxonomy comprises 14 separate components which may form part of self-management support interventions. The components were derived from an overview of systematic reviews of self-management for 14 long-term conditions, including type 2 diabetes. For each of these components, we have stated *a priori* assumptions of the likely impact of each component on patient workload and support for patient capacity. These assumptions have been presented and discussed with a patient advisory group, and feedback incorporated into these judgements (columns 3 and 4 of Table 1).

***Independent quantification of workload and capacity.*** The second phase will be to make a judgement of the overall ‘workload’ and ‘capacity support’ of each of the interventions. This stage of the process will be performed by separate reviewers, blinded to the study outcome. These reviewers will be provided with a summary of the intervention extracted during the previous, descriptive, phase.

**Table 1.** PRISMS taxonomy components and pre-specified assumptions of the impact on workload and capacity.

Component	Example(s) of use in T2DM	Likely impact on workload	Likely in impact on capacity
Information about condition and/or its management	Structured education programme on disease management	Provision of information itself **decreases** workload as reduces work of findings information. Could also be an **increase** if a programme requires attendance, appointments engagement etc.	**Increase** (better able to self-manage if better informed)
Information about available resources	Sources of emotional support, financial support and benefits, family networks, patient helplines	**Decrease** – reduces work of finding resources	**Neutral** (any increase in capacity likely to be the result of the resources themselves, which would fall into other categories in the taxonomy)
Provision of/agreement on specific clinical action plans and/or rescue medication	Specific advice on adjusting insulin dosage, or managing hypoglycaemia	**Decrease** - having explicit plans/guidelines reduced work of interpreting symptoms, seeking help/appointments if medication is needed etc.	**Increase** (Increases patient’s ability to self-manage: allows tailored, guided use of self-initiated medication. Reduced ambiguity and potential to increase confidence in self-management)
Regular clinical review	Regular clinical visits reviewing self- management strategies	**Increase** - demand of appointments, scheduling etc.	**Neutral** (clinical review *per se* unlikely to influence capacity overall. Interventions may improve capacity but this is likely to be due to aspects of the intervention falling into other categories (i.e. the content of the review determines the impact on capacity, and this can be classified under separate headings)
Monitoring of condition with feedback	Patients could send information about self-monitoring drug regimen and physiologic variables to physicians, who reviewed the data and sent personalized recommendations back to the patients	**Increase –** workload involved in collecting and sending data. Potentially **neutral** if feedback is useful and personalised and reduces need for appointments/seeking further information	**Increase** (provision of feedback likely to improve patient’s capacity to manage condition/symptoms. Further source of support.
Practical support with adherence	Reminders/follow-up following changes in medications. Weekly reminder telephone calls to perform foot care	**Decrease** - work of following regimen in shared/ supported.	**Increase** (provision of ‘practical’ support likely to improve adherence, leading on to potentially improved disease management)
Provision of equipment	Bag of supplies to enable foot care (containing soap, towel, socks, mirror, toenail clippers, lotion samples)	**Neutral or Increase** – depends on equipment – potentially increase required training, use of the equipment etc.	**Increase** (if useful for symptom control/functioning – e.g. aids use of colostomy)
Provision of easy access to advice or support when needed	Diabetes specific patient helpline. Designated person the patient can contact.	**Decrease** - ‘easy access’ key here: reduced work of seeking out advice	**Increase** (improvement in support/resources)
Training/rehearsal to communicate with health-care professionals	Community Support Workers from minority ethnic/deprived groups who provide advocacy and support communication with health- care professionals, attending an appointment with the person with LTC	**Decrease** - helps to facilitate interaction and communication, reducing workload. This weighting should be offset against any **increase** in workload if the training itself is particularly arduous.	**Increase** (specifically supportive intervention may impact social and psychological support, reduce anxiety of interactions with health-care professionals etc.)
Training/rehearsal for everyday activities	Supporting travel planning. Support explaining condition to employers, making adjustments etc.	**Decrease** - if seeks to aid/ease *everyday* functioning. This weighting should be offset against any **increase** in workload if the training itself is particularly arduous.	**Increase** (improvement in everyday function/activities as well as increased support for patient
Training/rehearsal for practical self-management activities	Practising foot care procedures	**Decrease** - training and support decreases the work involved in carrying out these activities (i.e. increases competence). This weighting should be offset against any **increase** in workload if the training itself is particularly arduous.	**Increase** (skills specifically aiming to allow self- management and facilitate control of/living with LTC)
Training/rehearsal for psychological strategies	Goal setting. Problem-solving strategies. Relaxation Reframing	**Decrease** – improving psychological strategies to cope with condition may reduce work involved in managing condition. This weighting should be offset against any **increase** in workload if the training itself is particularly arduous.	**Increase** (aiming to help adaptation, reduce psychological impact of condition)
Social support	Encouraging participants to interact and assess their own and their peers’ progress toward managing their diabetes by sharing ideas, advice, and support	**Neutral** – although ‘appointments’ but when this acts as social support then would probably not frame this as ‘work’	**Increase** (social support a key aspect of capacity within CCM)
Lifestyle advice and support	Monthly clinic visits with nutritionist providing advice to enhance physical activity and dietary intake	**Increase** or **neutral** depending on balance between supporting lifestyle intervention which the patient would already be trying to achieve and placing additional demands on patient to make further lifestyle changes.	**Increase** (positive dietary support (for example) likely to benefit overall patient experience/impact of LTC)

The reviewers will first describe the intervention components according to the PRISMS taxonomy, as well as recording the length of the intervention, the frequency of contacts, and additional factors (such as number of appointments) that may influence workload. They will then be presented with a summary of components categorised by the *a priori* judgements of the impact of components on workload and capacity.

The reviewers will then use this summary along with the written description of the intervention to make an overall assessment of the workload of the intervention and the support for capacity. Each construct (workload and capacity) will be graded separately using a visual analogue scale (ranging 0–100, 0 indicating a large reduction in workload or capacity, 50 indicating neutral impact on workload or capacity, and 100 indicating large increase in workload or capacity). This judgement will be based on the likely impact over the time-period of the trial follow-up.

An initial subset of studies will be graded and results of independent reviewers calibrated. Differences will be resolved by discussion prior to grading the remaining interventions.

The end-result of this process will be a score assigned to each trial estimating the ‘workload’ and ‘capacity support’ of the intervention. There will also be descriptive codes assigned to each intervention for the nature of the self-management support (according to the PRISMS taxonomy).

### Aim 2: Impact of intervention on clinical outcomes

Given the broad inclusion criteria of this systematic review, encompassing a wide range of self-management support interventions, we expect the included studies to be highly heterogenous in terms of intervention and study population. Our analysis is therefore primarily designed to explore sources of heterogeneity in the effectiveness of interventions, rather than produce a single summary estimate for self-management support interventions.

Interventions will be grouped first by mode of delivery according to the EPOC taxonomy of health system interventions ^23^. Depending on the number of included studies, these may be further sub-divided (e.g. remote interventions may be split into telephone interventions and telemonitoring interventions). Studies will also be grouped by outcomes assessed and length of follow-up. Where more than two studies are identified with similar mode of delivery, outcomes, and follow-up, these will be combined in a random-effects meta-analysis (anticipating a high degree of heterogeneity even within these groups of studies). Heterogeneity will be quantified using the I ^2^-statistic. Summary effects for each group of studies will be calculated along with 95% confidence intervals. Publication bias will be assessed using funnel plots.

Where there is either an insufficient number of studies assessing an outcome, or studies are too heterogeneous to allow for meta-analysis within subgroups, a narrative synthesis will be performed.

### Aim 3: Impact of workload and capacity support on outcomes

The impact of workload and capacity on the outcomes of interventions will be assessed using meta-regression. The scores assigned to each construct under Aim 1 will be used as covariates within a meta-regression model. The model will also include the modes of delivery (used under Aim 2) as additional covariates.

Secondary meta-regression analyses will be performed using the individual components of the PRISMS taxonomy (to explore the impact of individual components of self-management support) and using the frequency and intensity of the interventions.

## Discussion

This systematic review will provide a broad overview of self-management interventions for people living with type 2 diabetes. As self-management describes the tasks associated with living with a long-term condition, self-management support is a central component to the successful management of type 2 diabetes for all patients. The heterogeneity in the nature and efficacy of self-management support interventions is recognised and well-described. This review will therefore explore sources of this heterogeneity drawing upon patient-centred theoretical models of workload, treatment burden and capacity support ^6, 18– 20^.

The use of these theoretical models in this way draws upon previous systematic reviews using this framework to assess complex interventions. One review used this theoretical model to assess the impact of interventions aiming to prevent 30-day hospital readmissions, and showed that interventions that supported patients’ capacity for self-care were more likely to reduce readmissions ^25^. The Cumulative complexity model, along with the related theories of Normalisation Process Theory and the Theory of Patient Capacity ^18, 19^, have also been used to describe interventions using the Chronic Care Model in relation to multimorbidity ^26^. This review will draw upon these earlier applications of the cumulative complexity model to better understand how workload and capacity support impact on self-management support interventions for type 2 diabetes. Our hypothesis is that interventions are likely to be more effective if they balance the increased workload of self-management with support for patient capacity.

This explicit use of theoretical frameworks is important for two reasons. First, the Medical Research Council guidance for developing and evaluating complex interventions states that both development and evaluation of complex interventions demands strong theoretical foundations ^27^. Secondly, rising prevalence of multimorbidity and frailty among people living with type 2 diabetes ^10, 11, 15^, as well as established associations with lower socioeconomic status ^9^, mean that there is likely to be substantial variation in people’s capacity to complete the work involved in self-management. Therefore, understanding how these constructs relate to diabetes self-management specifically is likely to help to guide the development and reporting of future interventions.

A likely challenge with this systematic review will be the heterogeneity in included studies, particularly as the inclusion criteria are intentionally broad, and self-management interventions themselves are varied in their nature and content. This potential limitation is addressed by pre-specifying the methods which will be used to classify studies and identify those that may be reasonably combined in a meta-analysis. This approach draws upon two taxonomies: the EPOC taxonomy and the PRISMS taxonomy. The EPOC taxonomy is a well-established and refined taxonomy ^23^, relevant for classifying complex healthcare-system interventions ^28^. The PRISMS taxonomy more explicitly defined components of self-management support, and has also been used to group interventions in systematic reviews ^29^. This will allow a detailed and pre-specified classification of interventions, from which the appropriateness of combining studies in a meta-analysis can be judged.

As this project is a systematic review, ethical approval is not required.

The NHS Research for Scotland Primary Care Patient and Public Involvement group gave feedback on the initial conceptualisation of this project. The group were also involved in refining the judgements of how specific self-management components are likely to impact workload and patient capacity. These judgements will feed directly into the descriptive analysis described in the methods section as well as being used to inform the blinded assessment of workload and capacity.

Overall, we expect the results of this review to enhance our understanding of factors that influence a person’s ability to self-manage conditions like diabetes and thus to inform the design of future complex interventions to promote self-management.

## Data availability

Zenodo. Self-management interventions for Type 2 Diabetes: systematic review protocol focusing on patient workload and capacity support – PRISMA-P checklist. DOI: https://doi.org/10.5281/zenodo.5518081 ^30^

Data are available under the terms of the Creative Commons Attribution 4.0 International license (CC-BY 4.0).
