# Supplementary material for: Self-management interventions for Type 2 Diabetes: systematic review protocol focusing on patient workload and capacity support
Source: Wellcome Open Res. 2021 Oct 11;6:257. [Version 1] doi: 10.12688/wellcomeopenres.17238.1 (PMC9308000; doi:10.12688/wellcomeopenres.17238.1)
Supplement: Peer review history

# Peer review: Reviewer response for version 1

**DOI:** 10.21956/wellcomeopenres.19052.r51470

**Published:** 2022-07-22

**Authors:** Stephanie Jane Caroline Taylor

**Affiliations:** 1 Wolfson Institute of Population Health, Barts and The London School of Medicine and Dentistry, Queen Mary University of London, London, UK

This is a well designed protocol for a systematic review looking at self-management support for Type 2 DM and outcomes and considering the impact of the workload of the intervention components and the support interventions provide to the  capacity of the individuals with T2DM on outcomes. It is novel and theoretically driven. Consideration of intervention workload and individual's capacity might explain the heterogeneity in effects of self management support interventions and could lead to better understanding of this important area. 

 The assessments of intervention workload and potential for capacity for support are likely to be quite subjective and broad brush and I did wonder how accurate they will be. There is no mention of contacting authors of the original studies, for example. Nor is there any mention of identifying any qualitative studies published alongside the trials they include, which might throw more informed light on both intervention workload and support capacity. 

 I realise that this is not a conventional thing to do with a this sort of systematic review but it might be worth considering beyond this review as a a useful extension of the study. 

 Search strategy ends in 27 4 2021 (odd date not being a whole month), suggests already done but I would strongly advise they update it as by the time the results paper is sent for publication it will be more than 12 months old and most journals and reviewers want searches completed within 12 months of publication.

Is the study design appropriate for the research question?

Yes

Is the rationale for, and objectives of, the study clearly described?

Yes

Are sufficient details of the methods provided to allow replication by others?

Yes

Are the datasets clearly presented in a useable and accessible format?

Not applicable

Reviewer Expertise:

Public health and primary care, applied health services researcher

I confirm that I have read this submission and believe that I have an appropriate level of expertise to confirm that it is of an acceptable scientific standard.

# Peer review: Reviewer response for version 1

**DOI:** 10.21956/wellcomeopenres.19052.r46490

**Published:** 2021-10-29

**Authors:** Mireille Captieux

**Affiliations:** 1 Usher Institute of Population Health Sciences and Informatics, The University of Edinburgh, Edinburgh, UK

A very well thought out and clearly communicated protocol. Just a few points that might be helpful. 

 A small point, you first introduce workload in the introduction but it is not formally defined until the methods, so it might be easier for the reader if workload is explicitly defined after it is first mentioned.

 In terms of the methodology and allowing replication by others: 

- Will you be contacting study authors for more details about their interventions? e.g. studies may say they provide clinical reviews but the nature of the clinical review may not be clear. For example, this might be remote checking of HbA1c blood test that does not involve any work on behalf of the patient, in contrast to a face to face appointment where the patient's goals and progress are examined.  
- Your comparison is usual care/standard care. The relative difference in your main outcome (HbA1c) will depend on what constitutes "usual care". The heterogeneity in the control may also affect your assessment of the heterogeneity in the effectiveness of intervention. Usual care is unlikely to be the same across different countries with different healthcare systems. In some RCTs, usual care may be a self-management interventions in its own right e.g. information about condition and its management. Perhaps usual care may also need to be coded and thought given to whether authors are contacted for more details about what constitutes usual care. 
- Will you exclude any peer-reviewed studies on the basis of study quality/ risk of bias?
- Consider explicitly stating whether you will list all RCTs excluded at full-text

Aim 2:

- How will you define follow-up? This might be useful to think about particularly for RCTs where the distinction between the duration of active intervention and duration of follow-up with no active intervention is unclear. 

This is a well planned and ambitious piece of work, it would be good to see whether your patient advisory group will be informed of the findings and be involved in any recommendations for any change in patient care/guidelines.

Is the study design appropriate for the research question?

Yes

Is the rationale for, and objectives of, the study clearly described?

Yes

Are sufficient details of the methods provided to allow replication by others?

Partly

Are the datasets clearly presented in a useable and accessible format?

Not applicable

Reviewer Expertise:

Type 2 diabetes self-management from a primary care perspective

I confirm that I have read this submission and believe that I have an appropriate level of expertise to confirm that it is of an acceptable scientific standard.
